# Is Thy Secretary a Dog?

**Published:** 1917-12-01

**Authors:** 


*To the Editor of* THE HOSPITAL.

### "Is Thy Secretary a Dog?"

*To the Editor of* THE HOSPITAL.

SIR,—I do not know whether the paragraph in your issue of October 13, headed "They All Go Too Far" is to be taken as deliberate irony. If so, it is delightful, if rather cruel. I suppose that "much respected gentlemen" who are chairmen of large charitable institutions really do talk to each other like that; that they do in conversation with each other take it for granted that the relations between the chairman of a charitable institution and the secretary of the same (which institution, I may remark incidentally, is not the private property of either of them) are similar to those between a master and his "horse or dog." There is also something very engaging about the simplicity of the "much respected gentleman," who is quite convinced that if four practical experts in succession differ from him on hospital management, of which he knows perhaps nothing, this can only be due to "sauciness" on their part. It reminds one of the man who had been on five juries, and every time had found "eleven obstinate fellows who would not be convinced."

However, lest your contributor's irony should be found too subtle for the "much respected gentlemen" in question, I may venture to point out that in nine cases out of ten if the really competent and experienced secretary "begins to think and believe that he is running the hospital" it is not because he is "saucy," but because, as everyone acquainted with the practical working of such institutions knows, such may be the case. The hospital secretary, to be competent, should be a specialist in hospital management. Then on his success or failure depends principally the success or failure of the institution, however much the "much respected" chairmen may try to delude themselves to the contrary. The public credit of the hospital, the assessment of the grants under King Edward's Fund, etc., are based upon his work. The chairman, on the other hand, rarely knows anything of the minutiæ of such management, or could say how such minutiæ affect the working of the hospital. His activities are often confined to two or three visits, if as many, per week. Consequently a keen secretary, knowing his work and determined to make it a success, does undoubtedly often grow impatient and restive under the belated interference of men who have taken no trouble to acquaint themselves with the problems with which he has to deal, and whose haphazard suggestions would often, if adopted, undo the hard work and hard thinking of months, producing some disastrous muddle for which the secretary would, of course, have to take the blame.

In a word, the office of secretary to a hospital is by no means what your second "much respected". chairman calls it, a "soft job"; and it may perhaps relieve his mind to know that a capable and enterprising man is rarely "punished" in any mundane sense by losing it, but is, on the contrary, usually able to better his worldly position considerably, perhaps in the service of some commercial concern which does not dismiss its officers for showing capacity and activity, and could not survive for a year if it did. This fact, perfectly well known to both parties, tends also, no doubt, to create friction between energetic and even some secretaries of the best and the sort of chairman depicted in your dialogue.

But in my view the remedy for such trouble is not to put the secretary "in his proper place" along with the chairman's dog, but rather to encourage an able and energetic secretary to give effect to his policy so long as the success of that policy justifies him. That is the highly successful American method. In the hospitals of the United States the vigorous official is encouraged; the slack official is ruthlessly scrapped. British hospitals might be all the better for the enforcement of the American policy. And it may be opined, in the case of the hospital referred to in your paragraph, that the Council would have been better employed in stimulating, if possible, the senile tissues of its "much respected" but "no longer young" chairman than in dismissing four secretaries in succession apparently on account of their ability and zeal.

Meanwhile, what is the British Hospitals Association doing? Is it supposed to exist to protect the officers of hospitals against the consequences of the "horse or dog" view of their position? Might not one expect to hear from it some protest against the naked avowal of that view?—Yours, etc. OUTIS.

October 15, 1917.

### A Pharmacist's Contribution to Hospital Economy.

*To the Editor of* THE HOSPITAL.

